# Alteration in cerebral cortex thickness and structural covariance networks in patients with chronic prostatitis/chronic pelvic pain syndrome (CP/CPPS)

**DOI:** 10.3389/fneur.2026.1765343

**Published:** 2026-06-10

**Authors:** Huipeng Ren, Weixian Bai, Gengchen Ye, Ningkun Hong, Shan Li, Kun Zhang, Hang Su, Xiaohui Luo, Jianzhou Liu, Fengyan Yin, Jie Suo, Zhuanqin Ren, Hongzhe Tian, Xuan Niu, Ming Zhang

**Affiliations:** 1 Department of Medical Imaging, The First Affiliated Hospital of Xi'an Jiaotong University, Xi'an, China; 2 Department of Medical Imaging, Baoji Central Hospital, Baoji, China; 3 Xi'an Key Laboratory of Metabolic Disease Imaging, Xi'an No.3 Hospital, Affiliated Hospital of Northwest University, Xi'an, China; 4 Department of Urology, Baoji Central Hospital, Baoji, China

**Keywords:** chronic pelvic pain syndrome, chronic prostatitis, cortical thickness, graph theory, magnetic resonance imaging, structural covariance network

## Abstract

**Background:**

Recent studies have reported regional brain structural alterations in patients with chronic prostatitis/chronic pelvic pain syndrome (CP/CPPS). However, patterns of cortical thickness (CT) changes and the topological organization of structural covariance networks (SCNs) in CP/CPPS remain insufficiently understood. This study aimed to investigate CT alterations and SCN organization in CP/CPPS using structural magnetic resonance imaging and graph theoretical analysis.

**Methods:**

A total of 31 patients with CP/CPPS and 28 healthy controls (HCs) were included. All participants underwent three-dimensional T1-weighted magnetic resonance imaging. Cortical thickness was estimated based on the Destrieux atlas. CT-based SCNs were constructed using the Brain Connectivity Toolbox, and between-group differences in network topology were assessed using graph theoretical analysis.

**Results:**

Compared with HCs, patients with CP/CPPS exhibited regional cortical thickness alterations, including cortical thinning in the left planum temporale, left inferior frontal sulcus, left inferior part of the precentral sulcus, left subparietal sulcus, right lingual gyrus, right planum temporale, and right superior occipital sulcus and transverse occipital sulcus, as well as cortical thickening in the left superior frontal gyrus, left inferior temporal sulcus, and right short insular gyrus. Within patients, cortical thickness of the left subparietal sulcus was negatively correlated with PCS helplessness scores, whereas cortical thickness of the right short insular gyrus was positively correlated with NIH-CPSI quality of life impact scores. At the network level, CP/CPPS patients showed higher clustering coefficient (C_p_), longer characteristic path length (L_p_), and lower global efficiency (E_global_), suggesting increased network segregation and reduced integration. No significant differences were observed in local network metrics after correction for multiple comparisons; however, exploratory analyses (*p* < 0.01, uncorrected) suggested increased nodal degree in the left middle frontal gyrus and decreased nodal metrics in occipital regions.

**Conclusion:**

CP/CPPS may be associated with multi-level structural brain alterations, including regional cortical thickness changes and altered structural covariance network organization. These findings support the involvement of central nervous system structural abnormalities in CP/CPPS and provide preliminary insights into its neurobiological basis.

## 1 Introduction

According to the National Institutes of Health (NIH) classification, chronic prostatitis/chronic pelvic pain syndrome (CP/CPPS) is classified as prostatitis type III (1). The prevalence of CP/CPPS is approximately 4.5–9% (2), and the clinical symptoms mainly include pelvic pain, lower urinary tract symptoms, psychological problems (anxiety/depression), and sexual dysfunction (3), which seriously affect patients’ physical and mental health and quality of life. A variety of factors, including infectious, neurological, inflammatory, autoimmune, endocrine, and psychological factors, have been reported to be associated with CP/CPPS (4). More importantly, an increasing number of studies have shown that both top-down and bottom-up mechanisms are involved in the “central sensitization” process of chronic pain, which is accompanied by changes in the structure and function of the central nervous system (5, 6).

Recent neuroimaging studies have shown that patients with various chronic pain conditions exhibit changes in gray matter volume or cortical thickness in several brain regions, including the prefrontal cortex, parietal lobe, temporal lobe, occipital lobe, cingulate gyrus, insula, and hippocampus. These gray matter alterations show region-specific and volume/thickness differences that vary depending on the disease (7–10). These findings suggest that structural changes in brain regions involved in pain modulation and perception may play a critical role in the onset and progression of chronic pain. However, studies examining gray matter changes in the brains of patients with CP/CPPS remain limited. A comparative study involving 19 CP/CPPS patients and 16 healthy controls (HCs) detected no significant differences in gray matter volume (GMV) (11). Conversely, another investigation revealed a decrease in GMV in the anterior cingulate cortex of CP/CPPS patients relative to HCs, which was positively correlated with both the total and pain scores on the National Institutes of Health Chronic Prostatitis Symptom Index (NIH-CPSI) (12). These inconsistent results may stem from the high correlation between gray matter volume (GMV) and total intracranial volume (TIV), which complicates the correction process and may introduce bias in the GMV results (13, 14). In contrast, cortical thickness (CT) has a lower correlation with TIV and may provide more stable morphometric measurements. Moreover, CT measurements can detect subtle changes in the cerebral cortex (15, 16). Therefore, investigating subtle changes in CT in patients with CP/CPPS is crucial for identifying the key brain regions involved in neural regulation and for uncovering the core mechanisms driving CP/CPPS.

Chronic pain, however, is increasingly recognized as a network-level disorder. Simply identifying and analyzing cortical thickness changes in local brain regions is insufficient to fully understand the complex central pathological mechanisms underlying chronic pain and CP/CPPS. Using graph theory and statistical correlations derived from structural MRI metrics, such as cortical thickness, gray matter volume, and surface area, researchers have constructed topologically defined structural covariance networks (SCNs) to characterize brain organization (17, 18). This approach has shifted the research focus from dissecting specific brain areas to exploring inter-brain region correlations, and ultimately to understanding pathologies that affect the connectivity of the entire brain network (17). In recent years, SCNs have been increasingly applied in chronic pain research. Studies in chronic low back pain (19) and chronic migraine (20) have consistently reported decreased global efficiency, suggesting that chronic pain conditions share a common network-level signature of impaired information integration across the brain. However, to our knowledge, there appears to be a gap in the literature regarding the application of graph theory to analyze the topological alterations within SCNs in the brains of patients with CP/CPPS.

Unlike previous studies that primarily focused on gray matter volume in patients with CP/CPPS, the present study focused on CT, specifically in young and middle-aged patients. At the structural level, we hypothesized that CP/CPPS patients would show CT differences in brain regions involved in pain modulation and perception, including the prefrontal cortex, insula, temporal lobe, and occipital lobe. At the network level, we further hypothesized that the global and local topological properties of CT-based SCNs would differ between CP/CPPS patients and HCs.

Therefore, this study compared cortical thickness and CT-based SCNs between patients with CP/CPPS and HCs. We further analyzed group differences in SCN topological properties using graph theoretical methods to identify biologically relevant network characteristics of CP/CPPS and their potential pathophysiological implications.

## 2 Materials and methods

### 2.1 Subjects

This study was conducted between April 2020 and August 2021. Patients with chronic prostatitis/chronic pelvic pain syndrome (CP/CPPS) were recruited from the Urology Outpatient Clinic of Xi’an No. 3 Hospital. Age- and education-matched healthy controls (HCs) were enrolled during the same period. All participants were male. The study protocol was approved by the Medical Ethics Review Board of Xi’an No. 3 Hospital (Approval No. XSYL2020-21), and written informed consent was obtained from all participants in accordance with the Declaration of Helsinki. The inclusion criteria for the CP/CPPS group were as follows: (1) diagnosis of CP/CPPS according to the NIH classification criteria (1); (2) age between 18 and 40 years; (3) pain present for most of the time during the previous 3 months; (4) a total score of ≥12 and a pain subscore of >0 on the National Institutes of Health Chronic Prostatitis Symptom Index (NIH-CPSI) (21); (5) no history of deformity, infection, or tumor in the genitourinary system; (6) no history of neurological or psychiatric disorders, head trauma, brain tumor, or other chronic pain conditions; and (7) right-handedness. The inclusion criteria for the HC group were: (1) no history of neurological or psychiatric disorders, head trauma, brain tumor, or chronic pain disorders; and (2) right-handedness. Participants were excluded if they met any of the following criteria: (1) contraindications to MRI; (2) inability to complete the MRI examination; (3) history of deformity, infection, or tumor in the genitourinary system; (4) history of neurological or psychiatric disorders, head trauma, or brain tumor; or (5) chronic pain at other body sites. Ultimately, 31 patients with CP/CPPS and 28 HCs were included in the study.

### 2.2 Questionnaires

Prior to MRI scanning, all participants completed the National Institutes of Health Chronic Prostatitis Symptom Index (NIH-CPSI) and the Pain Catastrophizing Scale (PCS) to assess symptom severity and pain-related cognitive responses. Given the multidimensional nature of these scales, subscale scores were used in subsequent analyses.

The NIH-CPSI is a widely used instrument for evaluating symptoms in patients with CP/CPPS, including pain, urinary symptoms, and quality of life impact (21). It consists of 9 items divided into three subscales: pain (0–21), urinary symptoms (0–10), and quality of life impact (0–12), with a total score ranging from 0 to 43. Higher scores indicate greater symptom severity (22).

The PCS is a validated measure of pain-related catastrophic thinking (23). It includes 13 items assessing rumination, magnification, and helplessness. Each item is rated on a 5-point Likert scale ranging from 0 (“not at all”) to 4 (“all the time”), with higher scores indicating greater levels of pain catastrophizing. In HCs, the PCS was used to assess general pain-related catastrophic thinking rather than current pain symptoms.

### 2.3 Brain imaging acquisition

All participants underwent high-resolution three-dimensional T1-weighted MRI scanning on a 3.0-T Philips Achieva scanner using a 32-channel head–neck coil. The imaging parameters were as follows: repetition time (TR) = 8.2 ms, echo time (TE) = 3.8 ms, flip angle = 8°, field of view (FOV) = 240 × 240 mm, number of slices = 192, slice thickness = 1 mm, and voxel size = 1 × 1 × 1 mm^3^.

### 2.4 Data preprocessing and analysis

#### 2.4.1 Data preprocessing

All T1-weighted images were visually inspected to exclude obvious morphological abnormalities, motion artifacts, or other scanning artifacts. The original DICOM images were converted to NIfTI format using dcm2nii. Surface-based morphometry analysis was then performed using FreeSurfer version 7.4.1.^1^ The standard preprocessing pipeline included removal of non-brain tissue, automated Talairach transformation, gray and white matter segmentation, surface reconstruction, inflation of the cortical surface, registration to a common spherical atlas, and estimation of cortical thickness as the distance between the white matter and pial surfaces (24). As a surface-based morphometric measure, cortical thickness is generally considered to be less dependent on overall head size than volumetric measures. Cortical thickness maps were smoothed using a 10-mm full-width at half-maximum (FWHM) Gaussian kernel.

The cortical surface was parcellated into 148 regions according to the Destrieux atlas, with 74 regions in each hemisphere (25). The mean cortical thickness of all vertices within each region was then calculated and used as the regional cortical thickness value for subsequent analyses.

#### 2.4.2 Structural covariance network construction

Structural covariance networks (SCNs) were constructed based on regional cortical thickness using the Brain Connectivity Toolbox (BCT) implemented in MATLAB R2023b. Each brain region was treated as a network node. Pearson correlation coefficients (r values) of cortical thickness were calculated across subjects to construct group-level CT-based structural covariance matrices. Negative correlations were set to zero, and only non-negative values were retained for subsequent thresholding and construction of undirected binary networks.

To ensure complete network connectivity and avoid isolated nodes, each correlation matrix was thresholded across a sparsity range of 0.05–0.40 with a step size of 0.01, yielding a set of undirected binary networks. This sparsity range was selected based on established methodological studies (26) and commonly adopted practices in structural covariance network analysis (27), as it preserves network connectivity while reducing the inclusion of potentially spurious connections at higher densities. This thresholding procedure generated a series of undirected, unweighted binary networks for each group. Graph theoretical analysis was then performed to compare the topological properties of the SCNs between the CP/CPPS and HC groups.

To assess the robustness of our findings against atlas-dependent biases, we performed a sensitivity analysis using an alternative parcellation scheme—the Desikan-Killiany atlas (28)—which divides each hemisphere into 34 regions. Cortical thickness values were extracted using the same pipeline, and group comparisons were performed with identical statistical procedures (see Supplementary material for details).

#### 2.4.3 Graph analysis

Graph theoretical analysis included both global- and nodal-level network measures. The global network metrics analyzed were clustering coefficient (C_p_), characteristic path length (L_p_), normalized clustering coefficient (Gamma), normalized characteristic path length (Lambda), small-worldness (Sigma), global efficiency (E_global_), and local efficiency (E_local_). The nodal metrics analyzed were nodal degree, nodal betweenness centrality (BC), and nodal efficiency. Detailed definitions and mathematical formulas are provided in the Supplementary material.

### 2.5 Statistical analysis

Statistical analyses were performed using SPSS version 27.0 (IBM Corp., Armonk, NY, United States). Normality of the data distribution was assessed using the Shapiro–Wilk test. Continuous variables with a normal distribution were expressed as mean ± standard deviation (mean ± SD). Between-group comparisons of demographic and clinical variables (including age, years of education, and PCS scores) were conducted using independent-samples t-tests. A two-tailed *p* < 0.05 was considered statistically significant.

Group differences in cortical thickness across brain regions were assessed using independent-samples *t*-tests between the CP/CPPS and HC groups, without additional covariates. To correct for multiple comparisons across brain regions, Bonferroni correction was applied, with statistical significance set at *p* < 0.05 (Bonferroni-corrected).

Given the multidimensional nature of the PCS and NIH-CPSI, subscale scores were used in the correlation analysis to explore specific associations between regional cortical thickness and distinct symptom dimensions. Pearson correlation analysis was performed to assess the relationships between cortical thickness in regions showing significant group differences and clinical subscale scores within the CP/CPPS group. To account for multiple comparisons, false discovery rate (FDR) correction using the Benjamini–Hochberg procedure was applied, with statistical significance set at *p* < 0.05 (FDR-corrected).

For structural covariance network (SCN) analysis, between-group differences in global network topological properties were assessed using nonparametric permutation tests with 5,000 permutations. The area under the curve (AUC) for each network metric was calculated across the predefined sparsity range, and statistical significance was set at *p* < 0.05.

For nodal-level network metrics, FDR correction was applied to control for multiple comparisons, with statistical significance defined as *p* < 0.05 (FDR-corrected). In addition, exploratory results at a more stringent uncorrected threshold of *p* < 0.01 were also reported as hypothesis-generating findings.

## 3 Results

### 3.1 Demographic and clinical characteristics

A total of 59 subjects (31 patients with CP/CPPS and 28 HCs) were enrolled in this study. The demographic and clinical characteristics of the participants are summarized in Table 1. Significant differences were noted in total score (*t* = 2.641, *df* = 57, *p* = 0.019) and all subscores (Rumination, *t* = 2.694, *df* = 57, *p* = 0.016; magnification, *t* = 2.155, *df* = 57, *p* = 0.035; Helplessness, *t* = 1.751, *df* = 57, *p* = 0.045) of PCS between the two groups. No significant differences were observed between the two groups in terms of age (*t* = 1.431, *df* = 57, *p* = 0.081) and duration of education (*t* = 1.584, *df* = 57, *p* = 0.062).

**Table 1:** Demographic and clinical characteristics of the participants.

Variable	CP/CPPS	HCs	*t*-value	*p*-value
*N*	31	28		
Age (years)	31.87 ± 5.73	30.75 ± 3.53	1.431	0.081
Education (years)	15.06 ± 1.91	15.24 ± 2.06	1.584	0.062
Pain duration (months)	16.75 ± 19.03	-	-	-
NIH-CPSI				
Total score	21.03 ± 5.33	-	-	-
Pain	10.29 ± 2.69	-	-	-
Urinary symptoms	2.68 ± 3.17	-	-	-
Life quality impact	8.69 ± 2.56	-	-	-
PCS				
Total score	19.35 ± 8.22	13.46 ± 10.41	2.641	0.019^*^
Rumination	8.25 ± 3.54	6.01 ± 3.36	2.694	0.016^*^
Magnification	4.84 ± 2.13	3.23 ± 2.87	2.155	0.035^*^
Helplessness	6.26 ± 4.27	4.22 ± 2.21	1.751	0.045^*^

Data are presented as mean ± standard deviation (mean ± SD).

Between-group comparisons were performed using independent-samples *t*-tests (two-tailed).

All *t*-tests had df = 57. * *p* < 0.05 indicates statistical significance.

CP/CPPS, chronic prostatitis/chronic pelvic pain syndrome; HCs, healthy controls; NIH-CPSI, The National Institutes of Health Chronic Prostatitis Symptom Index; PCS, pain catastrophizing scale.

### 3.2 Between-group difference of cortical thickness

**Figure 1:**
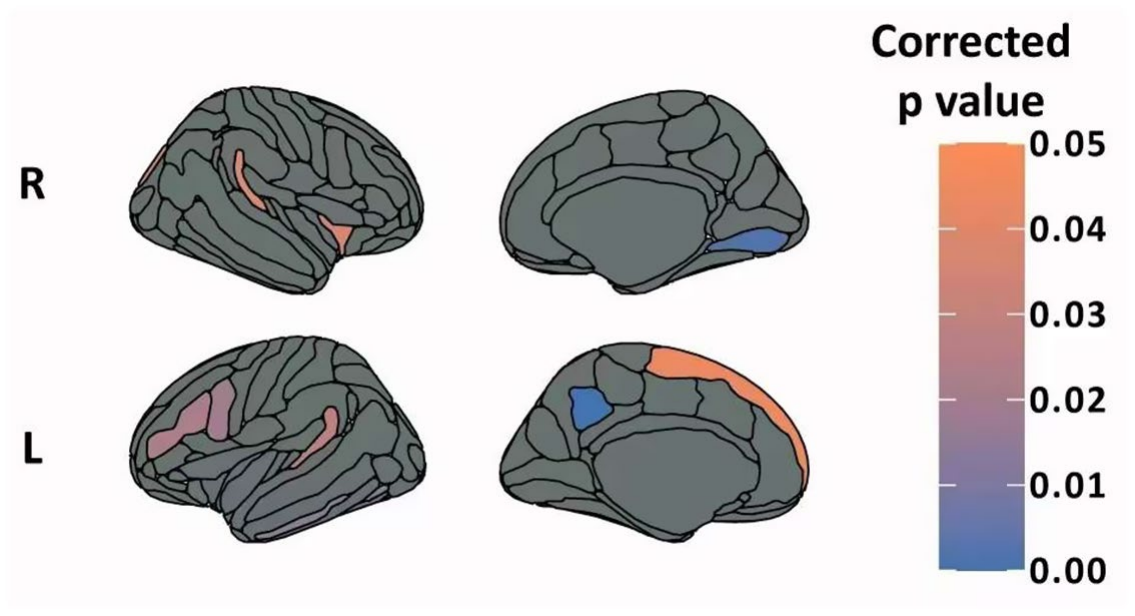
Brain regions showing significant differences in cortical thickness between patients with CP/CPPS and healthy controls. Regions with significant between-group differences are displayed on the cortical surface. The color bar represents Bonferroni-corrected *p* values. Warm colors indicate regions with increased cortical thickness in the CP/CPPS group, whereas cool colors indicate regions with decreased cortical thickness. R, right hemisphere; L, left hemisphere.

Group comparisons using independent-samples *t*-tests revealed that, compared with HCs, patients with CP/CPPS showed cortical thinning in several brain regions, including the left planum temporale, left inferior frontal sulcus, inferior part of the left precentral sulcus, left subparietal sulcus, right lingual gyrus, right planum temporale, right superior occipital sulcus and transverse occipital sulcus. In contrast, increased cortical thickness was observed in the left superior frontal gyrus, left inferior temporal sulcus, and right short insular gyrus in the CP/CPPS group (all *p* < 0.05, Bonferroni-corrected; Table 2; Figure 1).

**Table 2:** Brain regions showing significant differences in cortical thickness between CP/CPPS patients and healthy controls.

Region No.	Region name	CP/CPPS (mm)	HCs (mm)	*p*-value (Bonferroni-corrected)
L16	Left superior frontal gyrus	2.912 ± 0.172	2.859 ± 0.091	0.044
L36	Left planum temporale	2.456 ± 0.190	2.548 ± 0.129	0.028
L52	Left inferior frontal sulcus	2.197 ± 0.102	2.257 ± 0.115	0.023
L68	Left inferior part of the precentral sulcus	2.447 ± 0.142	2.518 ± 0.120	0.019
L71	Left subparietal sulcus	2.322 ± 0.140	2.429 ± 0.095	< 0.001
L72	Left inferior temporal sulcus	2.456 ± 0.149	2.386 ± 0.099	0.015
R18	Right short insular gyrus	3.415 ± 0.205	3.309 ± 0.227	0.036
R22	Right lingual gyrus	1.853 ± 0.116	1.940 ± 0.102	0.002
R36	Right planum temporale	2.427 ± 0.165	2.496 ± 0.113	0.040
R58	Right superior occipital sulcus and transverse occipital sulcus	2.112 ± 0.142	2.174 ± 0.150	0.039

Data are presented as mean ± standard deviation (mean ± SD). *p*-values were corrected using the Bonferroni method.

CP/CPPS, chronic prostatitis/chronic pelvic pain syndrome; HCs, healthy controls; L, left; R, right.

### 3.3 Associations between cortical thickness and clinical scores

**Figure 2:**
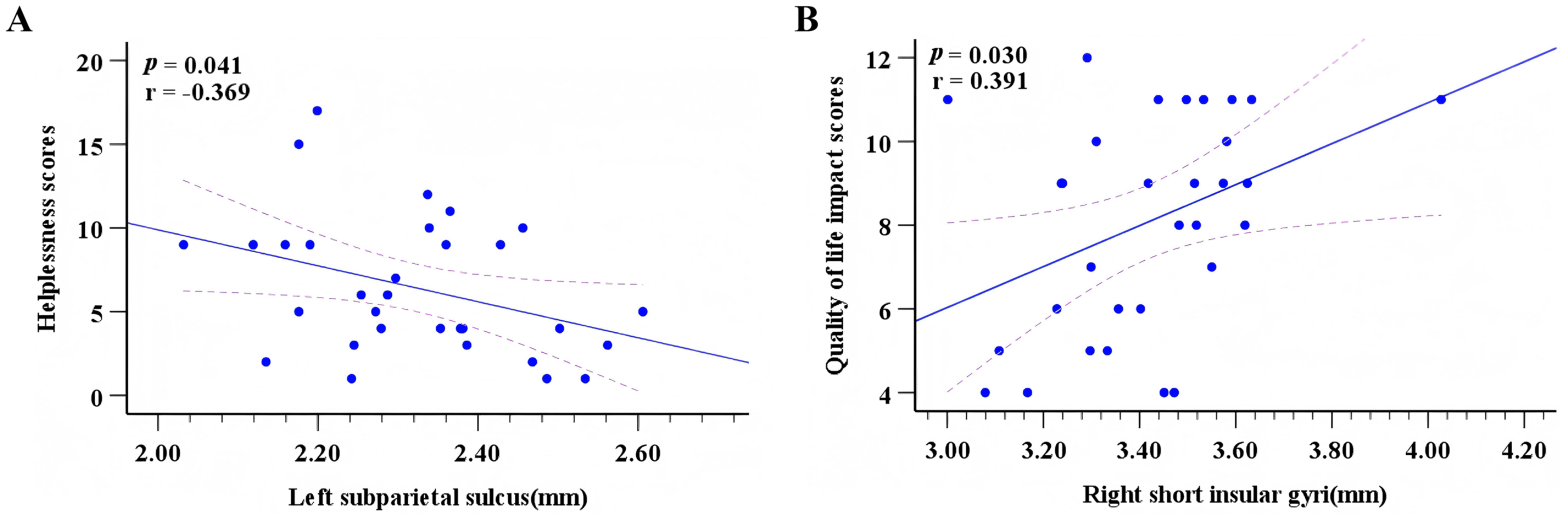
Significant correlations between cortical thickness and clinical scores in patients with CP/CPPS. The cortical thickness of the left subparietal sulcus was negatively correlated with the PCS helplessness score **(A)**, while the cortical thickness of the right short insular gyrus was positively correlated with the NIH-CPSI quality of life impact score **(B)**. Both correlations survived FDR correction.

Correlation analysis showed that, among the 10 brain regions with significant cortical thickness (CT) differences between groups, CT of the left subparietal sulcus was negatively correlated with the PCS helplessness score (*r* = −0.369, *p* = 0.041, FDR-corrected; Figure 2A), whereas CT of the right short insular gyrus was positively correlated with the NIH-CPSI quality of life impact score (*r* = 0.391, *p* = 0.030, FDR-corrected; Figure 2B). No other significant correlations were identified after FDR correction.

### 3.4 Structural covariance network

#### 3.4.1 Structural covariance matrices: CP/CPPS vs. HCs

**Figure 3:**
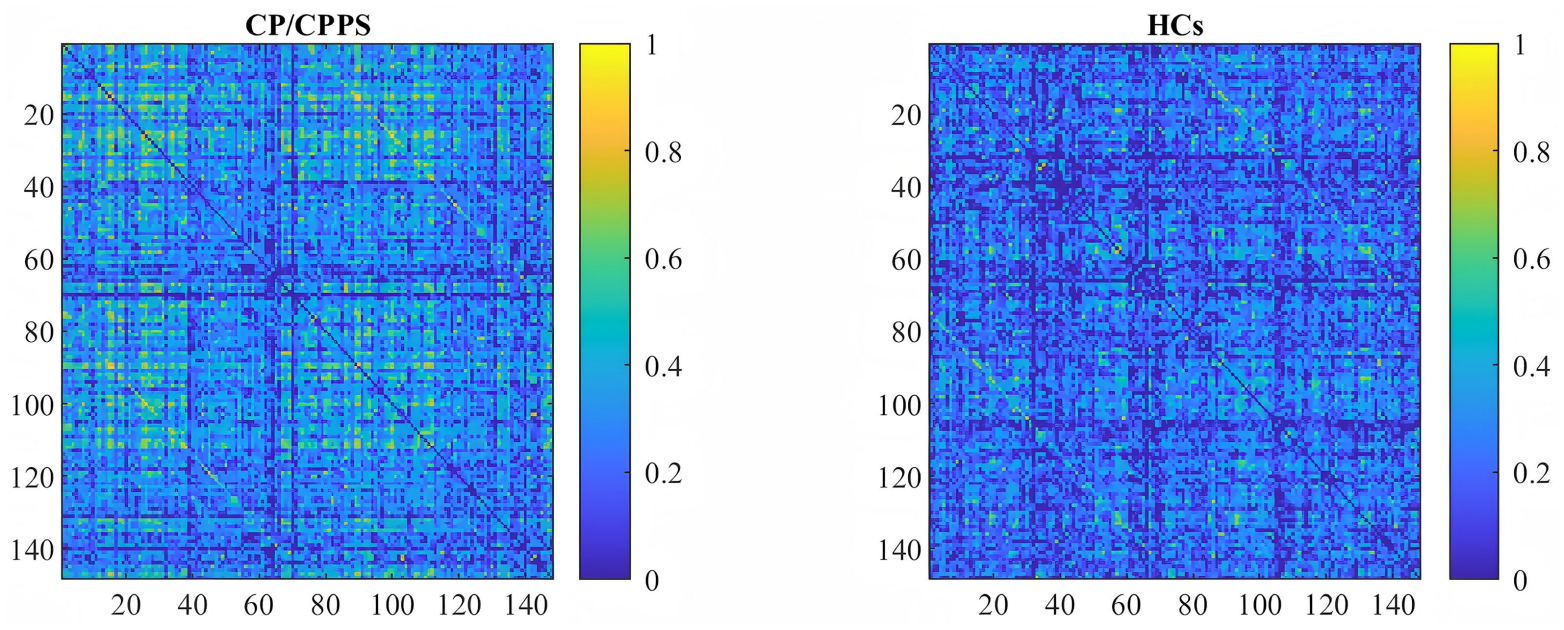
Correlation matrices of cortical thickness for each group. Each matrix represents the raw Pearson correlation coefficients (*r* values) of cortical thickness across 148 brain regions. The color bar indicates the strength of the correlation coefficients. Warmer colors indicate stronger positive correlations, whereas cooler colors indicate weaker correlations. Negative correlations were set to zero during matrix construction and are therefore not displayed. These matrices are shown for visualization purposes. CP/CPPS, chronic prostatitis/chronic pelvic pain syndrome; HCs, healthy controls.

Both groups exhibited a modular organization in their cortical thickness correlation matrices (Figure 3; 148 regions). Visual inspection suggested differences in correlation patterns within and between certain modules in the CP/CPPS group compared to HCs. It should be noted that these matrices are presented for visualization purposes only, and statistical differences were formally assessed using graph theoretical analysis. Therefore, graph theory analysis was subsequently performed to quantify these topological properties.

#### 3.4.2 Global graph measures: CP/CPPS vs. HCs

**Figure 4:**
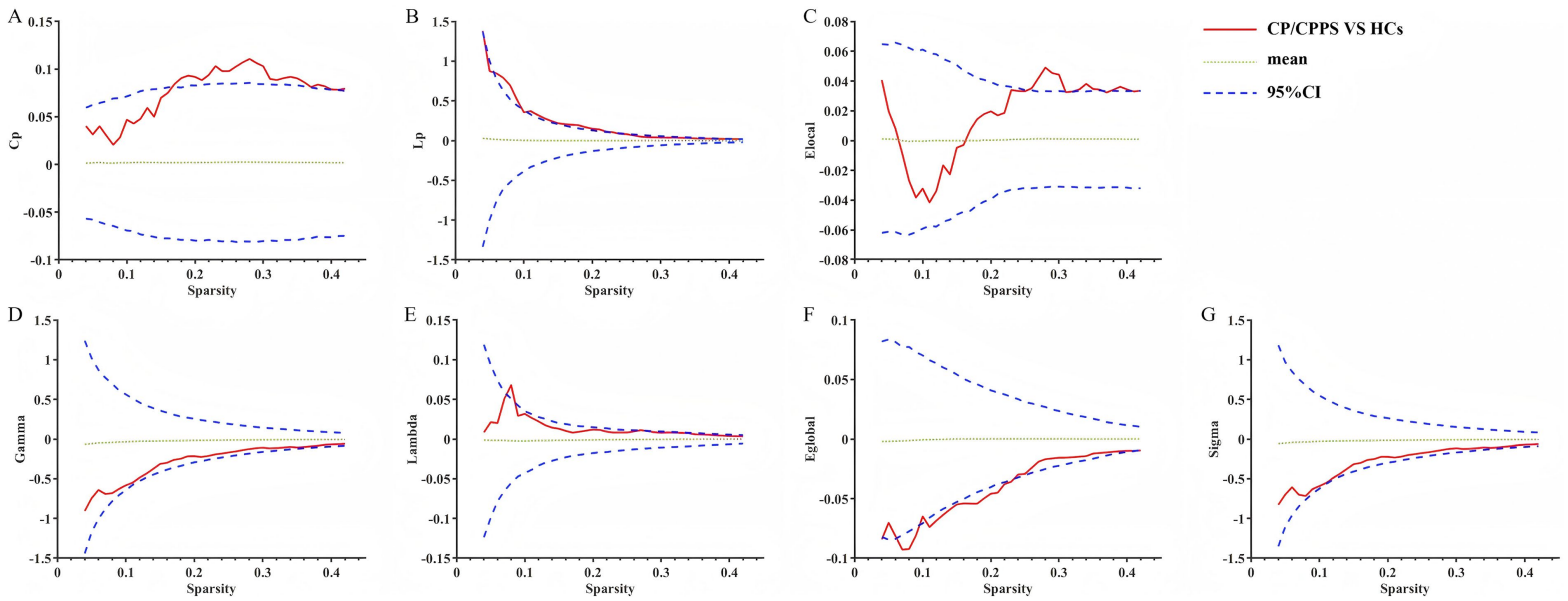
Between-group differences in global structural covariance network metrics across sparsity levels. **(A)** C_p_, **(B)** L_p_, **(C)** E_local_, **(D)** Gamma, **(E)** Lambda, **(F)** E_global_, and **(G)** Sigma. The *x*-axis represents network sparsity, and the *y*-axis represents the between-group difference in each network metric. Red solid lines indicate observed differences between CP/CPPS patients and HCs in the real structural covariance networks. Blue dashed lines indicate the 95% confidence intervals, and green dashed lines represent intergroup variability derived from random networks. Red lines located outside the 95% confidence intervals indicate statistically significant between-group differences (*p* < 0.05). Positive values indicate CP/CPPS > HCs, whereas negative values indicate CP/CPPS < HCs. CI, confidence interval.

**Figure 5:**
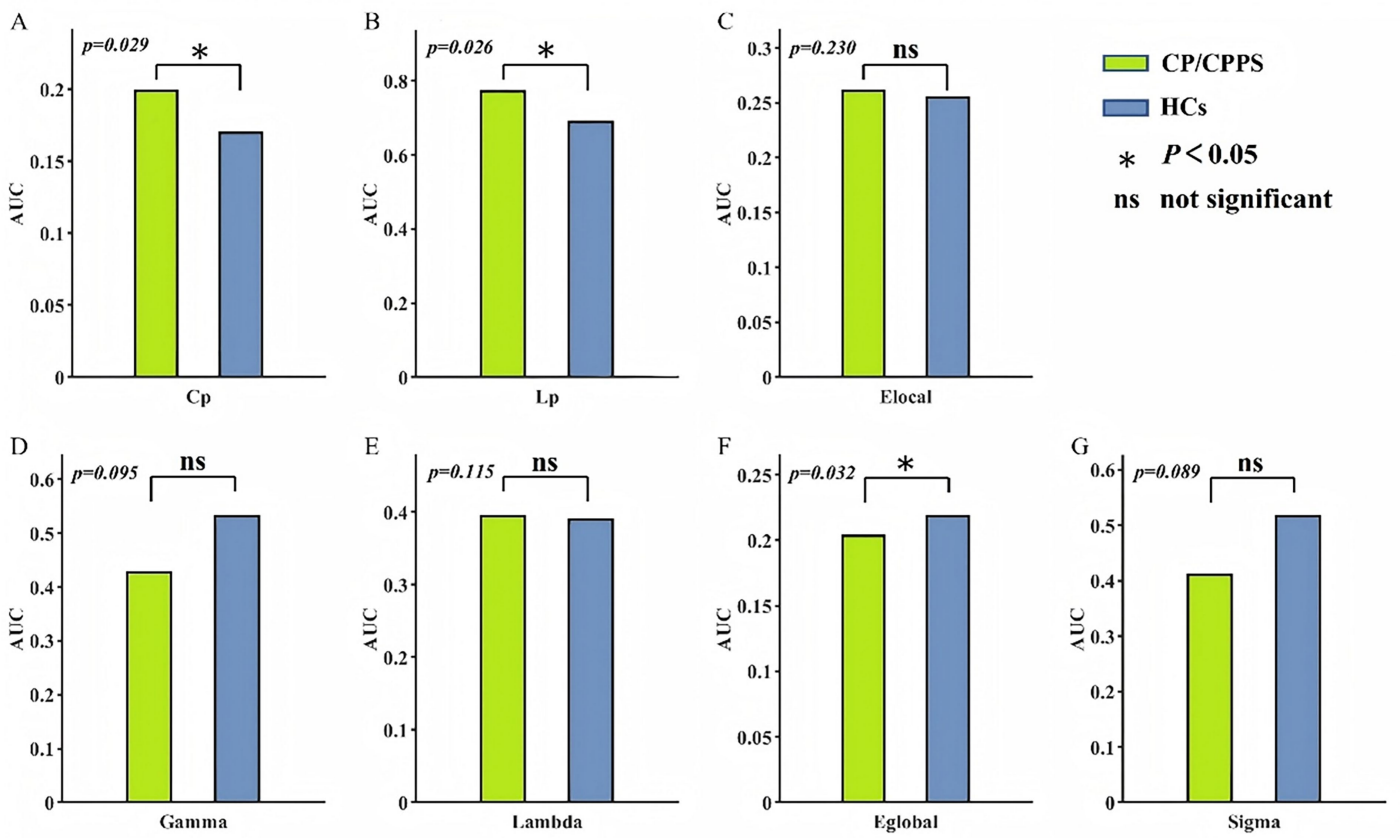
Between-group comparisons of global structural covariance network metrics based on area under the curve (AUC). **(A)** C_p_, **(B)** L_p_, **(C)** E_local_, **(D)** Gamma, **(E)** Lambda, **(F)** E_global_, and **(G)** Sigma. Bar plots represent the AUC values of each network metric across the predefined sparsity range in the CP/CPPS and HC groups. Between-group differences were assessed using nonparametric permutation tests.

At the global network level, patients with CP/CPPS showed significantly higher C_p_ and longer L_p_, as well as lower E_global_, compared with HCs (all *p* < 0.05; Table 3; Figures 4, 5), whereas no significant between-group differences were found in Gamma, Lambda, E_local_, or Sigma (all *p* > 0.05).

**Table 3:** Between-group comparisons of global structural covariance network metrics.

Graph measure	CP/CPPS (AUC)	HCs (AUC)	*p*-value
C_p_	0.199	0.169	0.029*
Gamma	0.426	0.531	0.095
L_p_	0.772	0.689	0.026*
Lambda	0.394	0.389	0.115
E_local_	0.261	0.254	0.230
E_global_	0.203	0.218	0.032*
Sigma	0.410	0.516	0.089

AUC, area under the curve. *p*-values were obtained from nonparametric permutation tests with 5,000 permutations. * *p* < 0.05 indicates statistical significance.

#### 3.4.3 Local graph measures: CP/CPPS vs. HCs

No significant between-group differences in nodal-level topological properties were observed after FDR correction (all *p* > 0.05).

At an exploratory level, analyses performed at a more stringent uncorrected threshold (*p* < 0.01) suggested potential alterations in nodal properties in several regions (Table 4). Specifically, CP/CPPS patients showed higher nodal degree in the left middle frontal gyrus, whereas lower nodal degree was observed in the right middle occipital sulcus and lunatus sulcus. In addition, lower nodal betweenness centrality was observed in the right superior occipital gyrus and right middle occipital sulcus and lunatus sulcus. Lower nodal efficiency was also observed in the left middle occipital sulcus and lunatus sulcus, right superior occipital gyrus, and right middle occipital sulcus and lunatus sulcus. These findings did not survive correction for multiple comparisons and should therefore be interpreted as suggestive rather than confirmatory.

**Table 4:** Between-group comparisons of nodal topological properties of structural covariance networks at an exploratory uncorrected threshold of *p* < 0.01.

Graph measure	Region No.	Region name	CP/CPPS	HCs	*p*-value
Nodal degree	L15	Left middle frontal gyrus	33.39	21.61	0.008*
	R57	Right middle occipital sulcus and lunatus sulcus	0.39	19.92	<0.001*
Nodal betweenness centrality (BC)	R20	Right superior occipital gyrus	4.78	273.99	<0.001*
	R57	Right middle occipital sulcus and lunatus sulcus	0.11	75.47	0.006*
Nodal efficiency	L57	Left middle occipital sulcus and lunatus sulcus	0.15	0.26	0.006*
	R20	Right superior occipital gyrus	0.18	0.27	0.006*
	R57	Right middle occipital sulcus and lunatus sulcus	0.06	0.25	<0.001*

Values represent nodal topological properties. Between-group differences were assessed using nonparametric permutation tests. No nodal-level findings survived FDR correction; therefore, the results shown here are reported at a more stringent uncorrected threshold (*p* < 0.01) and should be considered exploratory and hypothesis-generating. R, right; L, left; BC, betweenness centrality. * *p* < 0.01 indicates statistical significance.

## 4 Discussion

To our knowledge, this is the first study to employ a combination of surface-based morphometry and graph theoretical analysis to investigate structural alterations in patients with CP/CPPS, spanning from regional cortical thickness (CT) to global and nodal topological organization of structural covariance networks (SCNs).

Our principal findings are threefold. First, patients exhibited cortical thickness alterations in frontal, temporal, and occipital regions, including both thinning (e.g., inferior frontal sulcus) and thickening (e.g., superior frontal gyrus), suggesting potential regional structural remodeling. In addition, exploratory correlation analyses suggested potential associations between regional CT and clinical measures. Second, CP/CPPS patients exhibited altered global network topology characterized by increased clustering coefficient, longer path length, and reduced global efficiency, indicating increased network segregation and reduced integration. Third, exploratory nodal analyses (*p* < 0.01, uncorrected) provided preliminary evidence for increased nodal degree in the left middle frontal gyrus and decreased nodal metrics in occipital regions, although these findings did not survive multiple comparison correction. Collectively, these findings may suggest an association between CP/CPPS and structural reorganization of brain networks, although they should be interpreted with caution.

### 4.1 Regional cortical thickness alterations and clinical correlations

The observed pattern of CT alterations is consistent with the view that chronic pain may involve distributed brain networks related to sensory, affective, and cognitive processing (29). However, given the cross-sectional design of the present study, these findings should be interpreted as associative rather than causal.

The contrasting changes within the left dorsolateral prefrontal cortex (DLPFC)—specifically, thinning in the inferior frontal sulcus (L52) and thickening in the superior frontal gyrus (L16)—are particularly intriguing. The DLPFC is a key node in the top-down pain modulatory system, implicated in cognitive control, attention, and emotion regulation (30). Previous studies have reported cortical thinning in prefrontal regions in chronic pain, which has been interpreted as potentially related to long-term nociceptive input (31). Conversely, cortical thickening in nearby regions has been proposed to reflect possible neuroplastic adaptations (32, 33). Evidence from animal models of chronic pain has demonstrated that persistent nociceptive input can induce both maladaptive changes (e.g., synaptic pruning, dendritic retraction, glial-mediated phagocytosis) and adaptive/compensatory responses (e.g., dendritic arborization, synaptogenesis, astrocytic hypertrophy) within adjacent cortical columns (34, 35).

The bidirectional structural alterations observed in adjacent regions may suggest a complex interplay between maladaptive changes and adaptive neuroplastic remodeling within the left DLPFC in patients with CP/CPPS. However, these findings should be interpreted with caution given the cross-sectional nature of the present study, and future investigations incorporating longitudinal designs, multimodal imaging, and experimental models are warranted to further clarify these potential mechanisms.

Another finding was the increased cortical thickness of the right short insular gyrus and its positive association with the NIH-CPSI quality of life impact score. The insula plays an important role in interoception and in integrating sensory and affective aspects of pain (36, 37). The anterior insula, where the short gyri are located, has been implicated in affective processing and salience detection (38). The observed association may therefore suggest that structural variation in this region is related to the subjective experience of CP/CPPS. Similar patterns have been discussed in the context of chronic pain chronification (39, 40). However, given the cross-sectional nature of the present study, our findings do not allow direct inference about such mechanistic processes. This relationship should therefore be interpreted cautiously. In addition, the negative association between cortical thickness of the left subparietal sulcus and PCS helplessness scores represents an exploratory finding that should be interpreted with caution. This region is anatomically close to the precuneus/posterior cingulate cortex, a core node of the default mode network involved in self-referential processing (41–44). The observed relationship may suggest a potential link between structural variation in this region and pain-related cognitive processing. However, given the lack of correction for multiple comparisons, the cross-sectional nature of the study, and the absence of formal mediation or moderation analyses, these findings should therefore be considered preliminary and hypothesis-generating. Future studies with larger sample sizes, longitudinal designs, and more comprehensive behavioral assessments are needed to further investigate these relationships.

Structural alterations were also observed in temporal and occipital regions. Although these regions are not traditionally considered core components of pain-processing systems, previous studies have suggested their involvement in emotional regulation, multisensory integration, and attentional processes in chronic pain (8, 45–48). The present findings may therefore indicate that CP/CPPS is associated with widespread cortical alterations; however, the functional implications of these changes remain to be further clarified.

Taken together, these regional cortical thickness findings suggest that CP/CPPS may be associated with distributed structural alterations across multiple cortical areas. To further characterize whether such regional changes are accompanied by alterations at the network level, we next examined the global topological organization of structural covariance networks.

### 4.2 Global topological reorganization of structural covariance networks

Moving beyond regional findings, graph theoretical analysis suggested potential alterations in the global topological organization of SCNs in CP/CPPS patients. Specifically, increased clustering coefficient (C_p_) and characteristic path length (L_p_), together with decreased global efficiency (E_global_), may indicate a shift toward greater network segregation and reduced integration (49, 50).

The elevated C_p_ may reflect stronger local interconnections within clusters of brain regions, whereas increased L_p_ and reduced E_global_ may suggest less efficient communication across distant regions. Such a pattern has been interpreted in previous studies as reflecting altered balance between functional specialization and global integration (51–53). This imbalance between segregation and integration disrupts the optimal “small-world” topology that characterizes healthy brain networks, which typically support both specialized processing and efficient global communication (54, 55). Our findings are consistent with studies of other chronic pain conditions, such as chronic migraine and chronic low back pain, which also reported decreased global efficiency (19, 20). This convergence may suggest that altered network organization is a potential feature of chronic pain. However, it should be emphasized that these network metrics are indirect measures of structural organization, and their biological interpretation remains uncertain. Although reduced global efficiency has been linked to cognitive and affective processes in prior studies (56, 57), this interpretation should be considered tentative in the current context. Future studies incorporating neuropsychological assessments (e.g., executive function, attention, and emotion regulation tasks) are needed to examine whether alterations in global efficiency are associated with—or potentially mediate—cognitive–affective symptoms in CP/CPPS.

While these global metrics provide an overall view of altered network organization in CP/CPPS, they do not identify which specific regions may contribute most prominently to these changes. We therefore further considered nodal-level properties to explore potential region-specific alterations in network topology.

### 4.3 Nodal centrality changes: exploratory analysis

Although the nodal-level results did not survive FDR correction and should therefore be interpreted cautiously, analyses using a more stringent uncorrected threshold (*p* < 0.01) revealed several exploratory nodal differences that may provide preliminary clues for future studies. Specifically, increased nodal degree was observed in the left middle frontal gyrus, which is commonly considered part of the DLPFC. The increased nodal degree in the left middle frontal gyrus may suggest altered local network involvement of prefrontal regions in CP/CPPS, but this finding requires confirmation in larger samples.

In contrast, decreased nodal degree, betweenness centrality, and nodal efficiency were mainly observed in occipital regions, including the right superior occipital gyrus and bilateral middle occipital sulcus and lunatus sulcus. These occipital findings may suggest potential alterations in the network role of visual and sensory-integration regions in CP/CPPS. Although occipital regions are not traditionally considered core pain-processing areas, previous studies have reported structural or functional alterations in visual and sensory-related regions in chronic pain conditions (46–48). However, given that these findings did not survive FDR correction, they should be regarded as preliminary and hypothesis-generating rather than confirmatory.

Taken together, these findings suggest that focal cortical alterations in CP/CPPS may be accompanied by broader reorganization of network topology, providing a preliminary multi-level framework linking local structural changes, global network imbalance, and symptom-related brain alterations.

### 4.4 Clinical and translational relevance

Although the present cross-sectional study does not establish diagnostic or prognostic utility, the observed structural alterations may have potential translational relevance. Future studies with larger samples, longitudinal follow-up, and predictive modeling are needed to determine whether these imaging features could contribute to patient stratification, treatment monitoring, or prediction of clinical outcomes in CP/CPPS.

### 4.5 Limitations and future directions

Several limitations should be acknowledged. First, the cross-sectional design precludes causal inference or conclusions about the temporal evolution of the observed brain alterations; longitudinal studies are needed to clarify their direction and progression. In addition, the restricted age range (18–40 years) limits generalizability to younger or older populations.

Second, although the sample size is comparable to prior neuroimaging studies, it may still limit statistical power, particularly for detecting small effect sizes; therefore, exploratory nodal findings and other small-effect results should be interpreted cautiously and validated in larger cohorts.

Third, the use of the Destrieux atlas restricted the analysis to cortical regions and did not include subcortical structures that are also important in pain processing. Future studies integrating cortical and subcortical analyses may provide a more comprehensive characterization.

Fourth, correlation analyses were limited to regions showing significant cortical thickness differences, potentially overlooking associations in other areas. Whole-brain analyses with appropriate multiple-comparison correction are warranted in future studies. In addition, although subscale scores of the PCS and NIH-CPSI showed associations with cortical thickness, their potential mediating or moderating roles were not examined. Furthermore, total scores may offer complementary information regarding overall symptom severity, and this possibility should be explored in future studies.

Finally, the present study was based on structural MRI and structural covariance analysis. Multimodal approaches combining structural, functional, and clinical measures may further clarify the neural mechanisms underlying CP/CPPS.

## 5 Conclusion

This study suggests that CP/CPPS is associated with regional cortical thickness alterations and altered structural covariance network organization. Patients with CP/CPPS showed increased network segregation and reduced integration, suggesting disrupted global network topology. These findings support the involvement of central nervous system structural alterations in CP/CPPS and provide preliminary insights into its neurobiological basis. Future longitudinal studies are needed to clarify the temporal dynamics and clinical significance of these changes.

## Data Availability

The raw data supporting the conclusions of this article will be made available by the authors upon reasonable request and subject to institutional and ethical regulations.

## Footnotes

1
https://surfer.nmr.mgh.harvard.edu
