# Supplementary material for: Alteration in cerebral cortex thickness and structural covariance networks in patients with chronic prostatitis/chronic pelvic pain syndrome (CP/CPPS)
Source: Front Neurol. 2026 Jun 10;17:1765343. doi: 10.3389/fneur.2026.1765343 (PMC13290450; doi:10.3389/fneur.2026.1765343)
Supplement: Supplementary file 1 [file Data_Sheet_1.PDF]

Supplementary Materials

sensitivity Analysis Using the Desikan-Killiany Atlas

S1. Methods

To assess the robustness of our primary findings with respect to atlas selection, we performed a sensitivity analysis using the Desikan–Killiany atlas (Desikan et al. 2006) as an alternative parcellation scheme, which divides the cortex into 68 regions (34 per hemisphere).

Cortical thickness values were extracted using the same preprocessing pipeline as described in the main manuscript (see Section 2.4.1). Structural covariance networks (SCNs) were constructed following the same procedures as in the primary analysis. Briefly, Pearson correlation coefficients of cortical thickness were calculated across subjects to generate group-level association matrices, and Fisher's r-to-z transformation was applied to improve the normality of the correlation values.

The resulting matrices were thresholded over the same sparsity range (0.05–0.40, step = 0.01) to generate a series of undirected binary networks. Graph theoretical analyses were then performed to compute both global and nodal network metrics.

For global network analysis, the area under the curve (AUC) for each metric was calculated across the predefined sparsity range, and between-group differences were assessed using nonparametric permutation tests with 5,000 permutations.

For nodal-level analyses, between-group comparisons were also performed using permutation tests, and multiple comparisons were controlled using the false discovery rate (FDR) correction with a significance threshold of $p < 0.05$.

S2. Results

S2.1 Global Network Properties

As shown in Supplementary Table 1 and Supplementary Fig. 1, no significant between-group differences were observed in any global network metrics between CP/CPPS patients and HCs using the Desikan–Killiany atlas. Specifically, comparisons of the area under the curve (AUC) values based on nonparametric permutation tests revealed no significant differences in clustering coefficient (C_p), characteristic path length (L_p), normalized clustering coefficient (Gamma), normalized characteristic path length (Lambda), small-worldness (Sigma), global efficiency (E_{global}), or local efficiency (E_{local}) (all $p > 0.05$). No significant differences were observed across the entire sparsity range.

Supplementary Table 1. Between-group comparisons of global structural covariance network

metrics.

Graph Measure	CP/CPPS (AUC)	HCs (AUC)	*p*-value
C_P	0.026	0.027	0.726
Gamma	0.117	0.135	0.599
L_P	0.288	0.218	0.106
Lambda	0.102	0.098	0.625
E_{local}	0.035	0.037	0.733
E_{global}	0.023	0.029	0.146
Sigma	0.092	0.109	0.557

AUC, area under the curve. *p* values were obtained from nonparametric permutation tests with 5,000 permutations. Statistical significance was set at $p < 0.05$.

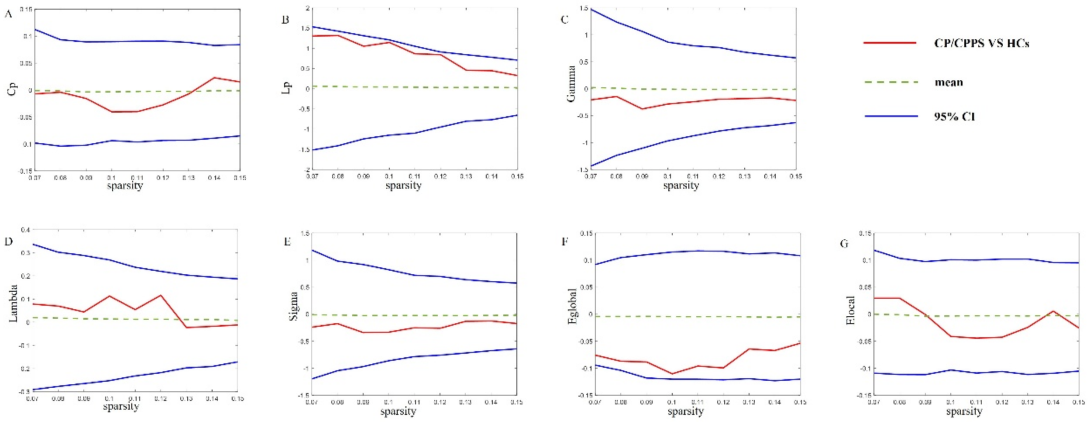

Supplementary Fig. 1. Between-group differences in global structural covariance network metrics across sparsity levels using the Desikan–Killiany atlas.

(A) C_p; (B) L_p; (C) Gamma; (D) Lambda; (E) Sigma; (F) E_{global}; and (G) E_{local}.
Red lines represent the observed between-group differences in the real structural covariance networks (SCNs), blue dashed lines indicate the 95% confidence intervals derived from permutation testing, and black lines represent the corresponding differences in randomized networks.
Red lines located outside the 95% confidence intervals indicate statistically significant between-group differences ($p < 0.05$). Positive values indicate CP/CPPS > HCs, whereas negative values indicate CP/CPPS < HCs.

S2.2 Nodal Network Properties

Nodal-level analyses similarly revealed no significant between-group differences in nodal properties (including nodal degree, betweenness centrality, and nodal efficiency) for any cortical region after FDR correction (all $p > 0.05$).

These findings are consistent with the results obtained using the Destrieux atlas, further supporting the robustness of the absence of nodal-level alterations in CP/CPPS under different parcellation schemes.

definitions of graph theoretical metrics

Graph theory analysis encompasses both global and local network analyses. It identifies seven key global network topological attributes and three principal local network topological attributes:

(1) clustering coefficient (C_p): the network metric C_p signifies the extent of connectivity and the degree of clustering among nodes within the network, a higher value of C_p suggests a denser local interconnectivity among the nodes (Rubinov and Sporns 2010).
(2) characteristic path length (L_p): the network parameter L_p is defined as the mean of the shortest paths between all pairs of nodes within the network, a lower Lp value indicates a more efficient system (Wu et al. 2021).
(3) normalized clustering coefficient (Gamma): Gamma is defined as the ratio of the Cp of the actual network to that of a corresponding random network, this ratio quantifies the separation function(Rubinov and Sporns 2010).
(4) normalized characteristic path length (Lamda): Lambda represents the ratio of the Lp of a real network to that of a random network, this metric quantifies the overall efficiency of the network's pathways(Rubinov and Sporns 2010).
(5) small-worldness (Sigma): Sigma index is the ratio of the Cp to the Lp, serving as a measure of the network's capacity to balance between integration and segregation(Bullmore and Sporns 2009).
(6) global efficiency (E_{glob}): E_{glob} represents the average reciprocal of the L_p between all pairs of nodes within a network. This measure reflects the overall efficiency of information transmission between the network's nodes(Zhang et al. 2020).
(7) local efficiency (E_{local}): E_{local} represents the inverse of the average shortest path length between neighboring nodes after the elimination of a node, thereby indicating the local information transmission capability of a network(He and Evans 2010).
(8) nodal degree: nodal degree denotes the count of connections a node has with other nodes within a network(He and Evans 2010).
(9) nodal betweenness centrality (BC): nodal BC is determined by the frequency with which a node lies on the shortest paths between all pairs of other nodes (Sporns 2013) .
(10) nodal efficiency: nodal efficiency is the local efficiency of a given node, which measures the communication efficiency between the first neighbor of the node when it is removed(He and Evans 2010).

References:

Bullmore, E., and O. Sporns. 2009. 'Complex brain networks: graph theoretical analysis of structural and functional systems', *Nat Rev Neurosci*, 10: 186-98.

Desikan, R. S., F. Ségonne, B. Fischl, B. T. Quinn, B. C. Dickerson, D. Blacker, R. L. Buckner, A. M. Dale, R. P. Maguire, B. T. Hyman, M. S. Albert, and R. J. Killiany. 2006. 'An automated labeling system for subdividing the human cerebral cortex on MRI scans into gyral based regions of interest', *Neuroimage*, 31: 968-80.

He, Y., and A. Evans. 2010. 'Graph theoretical modeling of brain connectivity', *Curr Opin Neurol*, 23: 341-50.

Rubinov, M., and O. Sporns. 2010. 'Complex network measures of brain connectivity: uses and interpretations', *Neuroimage*, 52: 1059-69.

Sporns, O. 2013. 'Structure and function of complex brain networks', *Dialogues Clin Neurosci*, 15: 247-62.

Wu, Z., Y. Gao, T. Potter, J. Benoit, J. Shen, P. E. Schulz, and Y. Zhang. 2021. 'Interactions Between Aging and Alzheimer's Disease on Structural Brain Networks', *Front Aging Neurosci*, 13: 639795.

Zhang, W., L. Guo, D. Liu, and G. Xu. 2020. 'The dynamic properties of a brain network during working memory based on the algorithm of cross-frequency coupling', *Cogn Neurodyn*, 14: 215-28.
